# Protocol: genetic transformation of the fern *Ceratopteris richardii* through microparticle bombardment

**DOI:** 10.1186/s13007-015-0080-8

**Published:** 2015-07-03

**Authors:** Andrew R G Plackett, Ester H Rabbinowitsch, Jane A Langdale

**Affiliations:** Department of Plant Sciences, University of Oxford, South Parks Road, Oxford, OX1 3RB UK

**Keywords:** Fern, Ceratopteris, Transformation, Transgenic, Protocol, Microparticle

## Abstract

**Background:**

The inability to genetically transform any fern species has been a major technical barrier to unlocking fern biology. Initial attempts to overcome this limitation were based on transient transformation approaches or achieved very low efficiencies. A highly efficient method of stable transformation was recently reported using the fern *Ceratopteris richardii*, in which particle bombardment of callus tissue achieved transformation efficiencies of up to 72%. As such, this transformation method represents a highly desirable research tool for groups wishing to undertake fern genetic analysis.

**Results:**

We detail an updated and optimized protocol for transformation of *C. richardii* by particle bombardment, including all necessary ancillary protocols for successful growth and propagation of this species in a laboratory environment. The *C. richardii* lifecycle comprises separate, free-living gametophyte and sporophyte stages. Callus is induced from the sporophyte apex through growth on cytokinin-containing tissue culture medium and can be maintained indefinitely by sub-culturing. Transgene DNA is introduced into callus cells through particle bombardment, and stable genomic integration events are selected by regeneration and growth of T_0_ sporophytes for a period of 8 weeks on medium containing antibiotics. Selection of T_1_ transgenic progeny is accomplished through screening T_1_ gametophytes for antibiotic resistance. In many cases sexual reproduction and development of transgenic embryos requires growth and fertilization of gametophytes in the absence of antibiotics, followed by a separate screen for antibiotic resistance in the resultant sporophyte generation.

**Conclusions:**

Genetic transformation of *C. richardii* using this protocol was found to be robust under a broad range of bombardment and recovery conditions. The successful expansion of the selection toolkit to include a second antibiotic for resistance screening (G-418) and different resistance marker promoters increases the scope of transformations possible using this technique and offers the prospect of more complex analysis, for example the creation of lines carrying more than one transgene. The introduction of a robust and practicable transformation technique is a very important milestone in the field of fern biology, and its successful implementation in *C. richardii* paves the way for adoption of this species as the first fern genetic model.

**Electronic supplementary material:**

The online version of this article (doi:10.1186/s13007-015-0080-8) contains supplementary material, which is available to authorized users.

## Background

Modern plant developmental genetics is often underpinned by the creation and study of transgenic lines to test gene function *in planta*. Stable genetic transformation of ferns has proved challenging in the past, and as such our understanding of fern development lags significantly behind almost all other land plant lineages despite adoption of the fern species *Ceratopteris richardii* for laboratory use [1]. The fern family is extremely diverse, comprising at least 10,000 species, and has a global geographic range across which species frequently compete successfully with angiosperms [2]. Given that some fern species are now recognized as having significant commercial and industrial value (e.g. the arsenic-hyperaccumulating fern *Pteris vittata* is currently being exploited for remediation of arsenic-contaminated soils [3]), interest in understanding fern developmental genetics is increasing.

**Figure 1:**
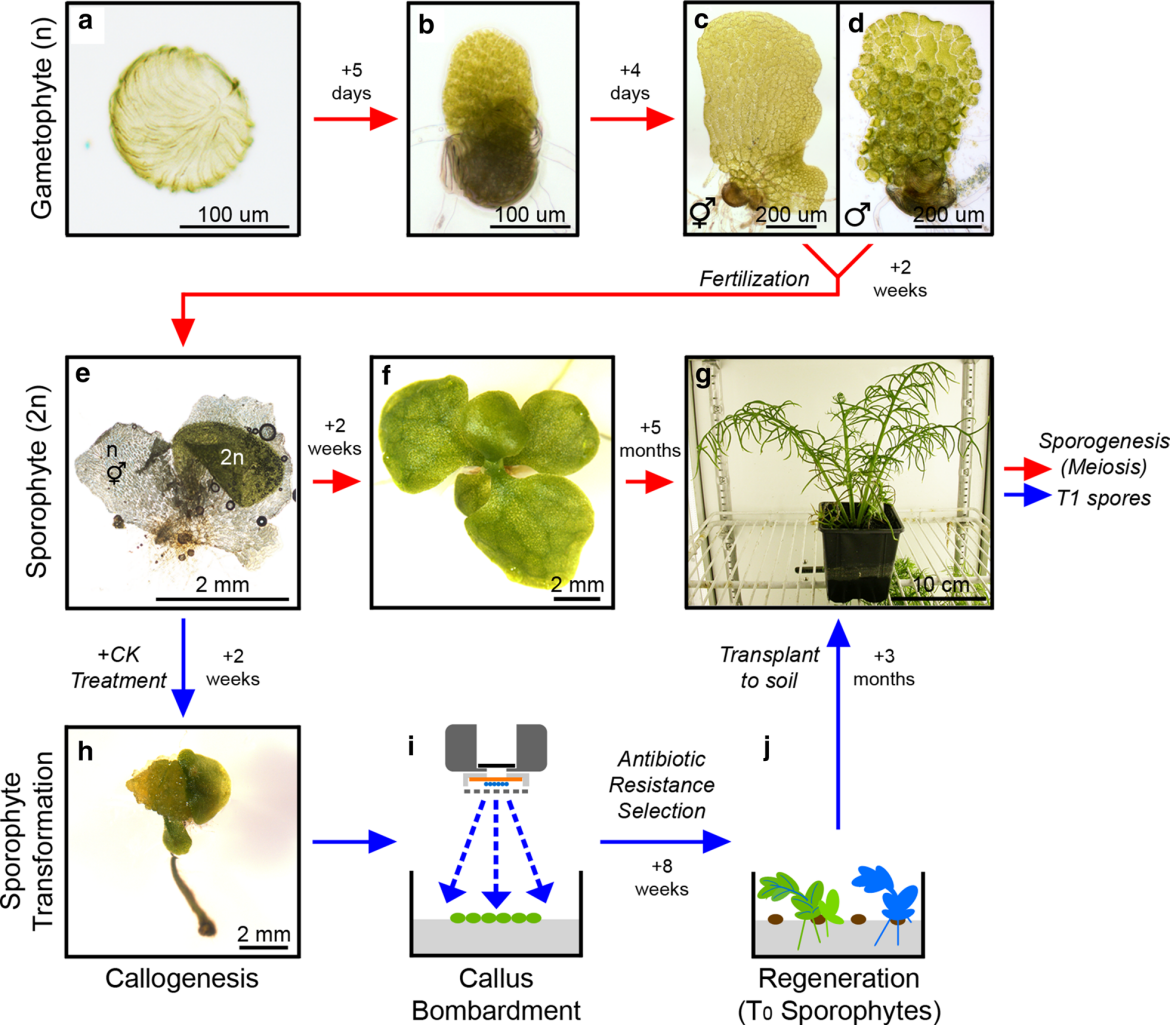
The lifecycle of *Ceratopteris richardii* and its relation to microparticle-mediated transformation. The *C. richardii* lifecycle comprises two separate, free-living stages, the haploid gametophyte (**a**–**d**) and the diploid sporophyte (**e**–**g**). Dispersed as haploid spores (**a**), these germinate and develop rhizoids and a two-dimensional thallus (**b**), assuming either hermaphrodite (**c**) or male (**d**) characteristics by sexual maturity (approximately 9–11 days after germination). Fertilization of gametes results in the development of sporophyte embryos on the hermaphrodite thallus (**e**), which continue to develop independently of the gametophyte, producing first simple fronds (**f**) and then fronds of increasing size and complexity until the mature, spore-bearing stage is achieved (**g**). Genetic transformation of *C. richardii* is performed on the sporophyte (**h**–**j**). Young sporophytes are treated with cytokinin (CK) to induce callus tissue at the shoot apex (**h**) in place of new fronds (**f**) [9]. This callus is isolated in tissue culture and transformed through particle bombardment (**i**) of plasmid DNA carrying the transgene of interest and a linked antibiotic resistance marker. Stable transgene integrations into the *C. richardii* genome are identified by screening for antibiotic resistance during sporophyte regeneration (**j**): antibiotic is added to the media, killing untransformed callus cells. Regenerated transformant shoots (designated T_0_) can then be grown to maturity and transgenic T_1_ spores (the progeny) harvested.

Ferns demonstrate an unusual lifecycle in that both the gametophyte (Figure 1a–d) and sporophyte stage (Figure 1e−g) grow as independent organisms, presenting a wide range of target stages for transformation. Whilst direct uptake of both RNA and DNA by germinating haploid fern spores [4–6], and particle bombardment of developing gametophytes [7] were successfully demonstrated as methods of transient transformation, until recently a viable method for the creation of stable transgenics proved elusive. *P. vittata* and a close relative of *C. richardii*, *C. thalictroides*, were successfully transformed by *Agrobacterium tumefaciens* infection of germinating spores [8], but transformation efficiencies were very low (0.053 and 0.03%, respectively). An alternative method utilizing particle bombardment of sporophyte-derived callus tissue (Figure 1h–j) [9] has since been developed, achieving more practical transformation efficiencies for both *C. thalictroides* and *C. richardii* (up to 86 and 72%, respectively). Since its first publication this method has undergone further testing and optimization to streamline the procedure for routine laboratory use with *C. richardii*. Here we present a detailed step-by-step protocol of this optimised method to allow adoption of this transformation technique by researchers not currently familiar with handling ferns.

## Materials

### Reagents

- *Ceratopteris richardii* gametophytes and sporophytes are grown in tissue culture on C-fern nutrient [10] 1% agar medium, pH 6.0. This media can be bought commercially or prepared from stock solutions as described in [11] (Additional file 1). Sterile tissue culture plates of 90 mm diameter are used, requiring 25 ml of medium per plate.
- Callus and regenerating sporophytes are grown in tissue culture on 1× Murashige and Skoog (MS) 2% sucrose 0.7% agar medium, pH 5.8 (MS-sucrose medium). Sterile tissue culture plates of 90 and 50 mm diameter are both used, requiring 25 and 15 ml of medium per plate, respectively.
- Sporophytes are grown to maturity on soil (potting compost, e.g. Levingtons F3 soil mix).
- Callus is generated and maintained by application of the cytokinin (CK) analogues 6-benzylaminopurine (BAP) or kinetin (KT) in MS-sucrose medium at 5 μM final concentration. Selection for stable transformants is carried out adding antibiotic (hygromycin B or the kanamycin analogue G-418) to MS medium at the required concentration (Table 1). Stocks of 5 mM BAP, 5 mM KT, 40 mg/ml hygromycin B and 50 mg/ml G-418 can be prepared in deionised sterile water, filtered through a 0.2 μm filter and stored indefinitely at −20°C.

**Table 1:** Antibiotic selection conditions for *C. richardii* transformation

Antibiotic	Concentration (μg/ml) required for screening		
	Callus	Gametophyte	Sporophyte
Hygromycin B	40	20	20
G-418	50	20	20

Final concentrations of antibiotic (as specified) required in growth media for successful identification of transgenic tissues from callus, gametophyte and sporophyte populations in tissue culture. Hygromycin B and G-418 concentrations were determined empirically ([9]; Additional file 2).
- Callus is bombarded using tungsten microparticles (average diameter ≈ 1.3 μm) labelled with 5 μg of purified plasmid DNA at a concentration of 1 μg/μl. Microparticle labelling requires sterile 50% glycerol, 2.5 M CaCl_2_ and 0.1 M spermidine (each sterilised through a 0.2 μm filter), and 70 and 100% ethanol (EtOH).
- *Ceratopteris richardii* is propagated by spores, which are available commercially and from various research laboratories. This protocol is optimised for use with the Hn-n strain, and has not been trialled on other strains.
- Spores are sterilised prior to use with a mixture of sodium hypochlorite solution (2% chlorine) and Tween-20 (0.1% v/v).
- Cellophane discs (90 mm diameter) (AA Packaging Ltd., UK).
- Filter paper (90 mm diameter).
- Sterile 90 mm diameter and 50 mm diameter tissue culture plates.
- Sterile 2 ml microcentrifuge tubes.
- Sterile Pasteur pipettes.
- General reagents for tissue culture e.g. 70% EtOH, parafilm, micropore tape.

### Equipment

- Growth of *C. richardii* requires continuous high humidity and high temperature. It is recommended that controlled environment facilities are used that are capable of maintaining constant ≈ 85% humidity with a long day (LD) growth regime of 16 h light/8 h dark, 28°C/28°C. Controlled humidity is not strictly required for growth of gametophytes, callus or regenerating sporophytes in tissue culture.
- Biolistic particle delivery system (e.g. Bio-Rad PDS-1000/He™) and associated reagents.
- Class 1 laminar flow tissue culture cabinet.
- Sterile forceps. Whilst watchmakers’ forceps are recommended for the transfer and manipulation of sporophytes during tissue culture, bayonet forceps are recommended to make the sterile transfer of callus tissue easier.
- Platform vortexer.
- Centrifuge.

## Protocol

The protocol for transformation of *C. richardii* comprises the following steps: (1) generation of callus tissue from the sporophyte apex, (2) transgene introduction via particle bombardment, (3) selection of stable transformation events and regeneration of T_0_ sporophytes through antibiotic selection, and (4) establishment of transgenic lines from the T_1_ progeny. This last stage is necessary because in proof-of-principle experiments, T_0_ transgenic shoots were frequently found to be chimeric [9] and thus not all T_1_ progeny will carry the desired transgene. Whilst this protocol was found to achieve robust transformation efficiencies under a range of experimental conditions in our hands, the majority of steps detailed in this protocol are involved with tissue culture, and many are performed under sterile conditions. Therefore, prior practical experience of tissue culture techniques will make adoption of this protocol easier, and presumably improve the results obtained using this protocol in the short term. The time intervals given in this protocol reflect a practiced user in an environment where the system is already established.

### Plasmid design: antibiotic resistance selection markers

In addition to the desired transgenic construct, plasmids for transformation of *C. richardii* require a linked antibiotic resistance selection marker under a constitutive promoter. *C. richardii* callus is susceptible to hygromycin B [9] and the kanamycin analogue G-418 (Additional file 2); the genes hygromycin B phosophotransferase (*HygR*) [9] and Neomycin phosphotransferase II (*NPTII*) (Additional file 3) can both be used as selection markers. The commonly-used viral 35S promoter drives sufficient expression in *C. richardii* for use with a selectable marker [9]. Similarly, the nopaline synthase (*nos*) promoter (295 bp) in the Gateway binary vector system [12] is sufficient to confer resistance in test transformations (Additional file 3). However, a truncated *nos* promoter variant (184 bp) from the pART27 vector [13] failed to regenerate T_0_ shoots under antibiotic selection (Additional file 3). As such, not all existing plant vectors can be expected to function in *C. richardii*. Where possible, it is recommended that expression tags be included with the target transgene to allow rapid determination of functional transgene expression in T_0_ shoots and T_1_ transgenic lines: plasmid shearing during particle bombardment means that the presence of a resistance marker is not a guaranteed indicator of a linked transgene [14].

#### A. Callogenesis and callus maintenance

Callus tissue is generated from young sporophytes. Unlike seed plants, ferns propagate through haploid spores (Figure 1a), thus requiring germination and growth of the gametophyte phase in sterile tissue culture to obtain sporophytes for callogenesis. Initial stocks of sterile spores can be obtained commercially, or previously harvested spores can be sterilised prior to germination. Spores are stored dry at room temperature. A 20 mg aliquot of dry spores typically provides sufficient material for up to eight plates, although sufficient stocks of callus for transformation can ultimately be obtained from a single plate of spores.

1. Culture gametophytes from spores under sterile conditions on 90 mm diameter C-fern medium tissue culture plates (see detailed protocols in Additional files 1, 4) until sexually mature (Figure 1c, d; typically 9–11 days after sowing).
2. Add 4–5 ml sterile water to each plate to fertilize the gametophyte population and induce sporophyte development. Swirl the plate gently to distribute the water evenly, then reseal and return to incubator. To maximise the frequency of fertilization, repeat this step after 3–4 days. Subsequently, maintain high humidity within each plate by adding 2 ml sterile water every 4–5 days.
  *Note* If fertilization is successful, sporophytes typically become visible approximately seven days after fertilization, growing on the gametophyte thallus and distinguishable as a darker green colour (Figure 1e).
3. Sporophytes are suitable for callus induction once they have developed a visible shoot and root, typically between 10 and 14 days after fertilization (Figure 2a). Induce callus by transferring whole individual sporophytes to 1 × MS 2% sucrose 0.7% agar plates (pH 5.8) containing 5 μM BAP (MS-BAP medium). Seal the plates with micropore tape and incubate under 28°C LD growth conditions. After 10–14 days, callus tissue should become visible at the shoot apex in place of new fronds (Figure 2b).
  *Note* In our hands *C. richardii* callus tissue developed poorly on C-fern medium [9], and as such it should not be used for callus induction or maintenance. The inclusion of auxin in the growth medium [9] was found to be unnecessary for both induction and maintenance of callus tissue (Additional file 5). Healthy callus suitable for transformation is typically emerald green in colour and friable.

**Figure 2:**
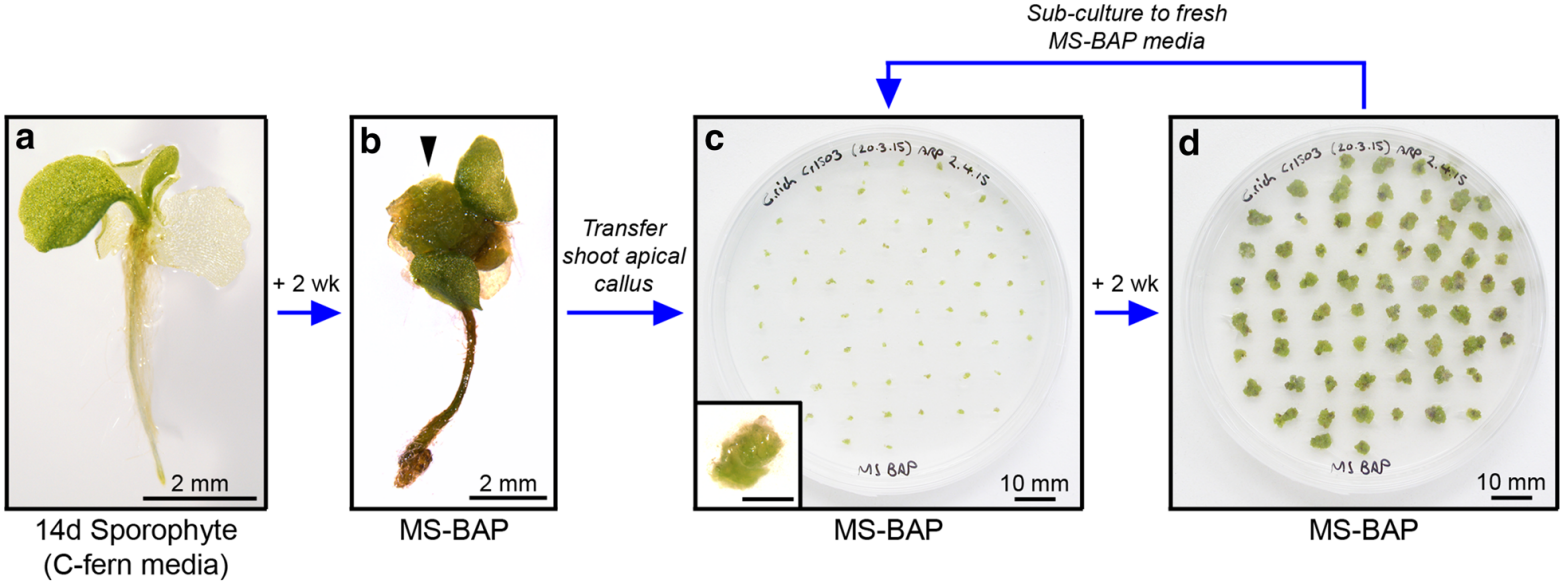
Callogenesis and callus maintenance. Callus tissue is induced from young sporophytes (**a**). Growing sporophytes on 1× MS 2% sucrose 0.7% agar medium (pH 5.8) containing 5 μM BAP (MS-BAP) causes development of callus at the shoot apex within 2 weeks (*arrowhead* **b**). Callus tissue is then removed from the progenitor sporophyte using forceps and transferred to fresh MS-BAP medium (**c**). *Inset* shows individual callus piece, *scale* 1 mm. Callus tissue on MS-BAP maintains an undifferentiated fate and will grow independently. After 2 weeks (**d**) callus is typically large enough for bombardment and/or sub-culturing to fresh medium to maintain callus stocks for future bombardments.
4. Using sterile watchmakers’ forceps, excise small pieces of callus tissue from the apex and transfer to fresh MS-BAP plates (Figure 2c). Incubate under under 28°C LD growth conditions.
  *Note* Callus plates do not require the addition of water to maintain callus growth.
5. To maintain stable callus stocks in tissue culture, transfer small pieces from existing plates to fresh MS-BAP medium (sub-culturing) every 2 weeks (Figure 2d). At 2 weeks a single plate of callus is typically sufficient to prepare two bombardment plates (see step B1).
  *Troubleshooting* If callus colonies become too large then tissue differentiation can occur. If the initial callus population shows strong signs of differentiation after separation from the sporophyte, sub-culture the callus after 1 week to maintain high penetration of BAP into the tissue.
  *Note* Callus stocks can be maintained in tissue culture for over a year and remain competent for transformation [9], but in our experience the quality of regenerated T_0_ shoots that are recovered from transformation degrades over time with ageing callus stocks. To maintain maximum quality it is recommended that a fresh callus stock be generated every 3–4 months.

#### B. Callus transformation and regeneration of T_0_ transgenic sporophytes

*Ceratopteris richardii* callus tissue is transformed by particle bombardment. Proof-of-principle experiments found that some regenerated transgenic T_0_ shoots either do not carry or do not express the desired transgene [9], and these cannot be distinguished from fully-transgenic shoots when transplanting to soil. The bombardment and regeneration conditions outlined below have been optimised through tests using a *35S::GUS* transformation marker to increase the proportion of genuinely transgenic sporophytes within the regenerated population (see Additional file 6). All of the following steps should be carried out under sterile conditions in a laminar flow cabinet, unless stated otherwise.

1. Transfer callus from MS-BAP stock plates to 1xMS 2% sucrose 0.7% agar (pH 5.8) 5 μM KT (MS-KT) 50 mm bombardment plates (see Additional file 1). Callus pieces should be arranged very densely to create a compact circular target for bombardment approximately 30 mm in diameter (Figure 3a).
  *Note* Bombardment plates can be prepared up to 2 days prior to bombardment, with further callus growth during this time increasing target density, but in this scenario plates should be sealed with parafilm and incubated at 28°C under LD conditions prior to bombardment.
  *Note* Callus should not be bombarded or regenerated on media containing BAP, which blocks subsequent shoot regeneration even 6 weeks after BAP treatment has been removed (Additional file 6). Although not essential for successful regeneration, including KT in the media during bombardment and for 2 weeks afterwards marginally increases regeneration efficiency and the final size of regenerated shoots (Additional file 6).

**Figure 3:**
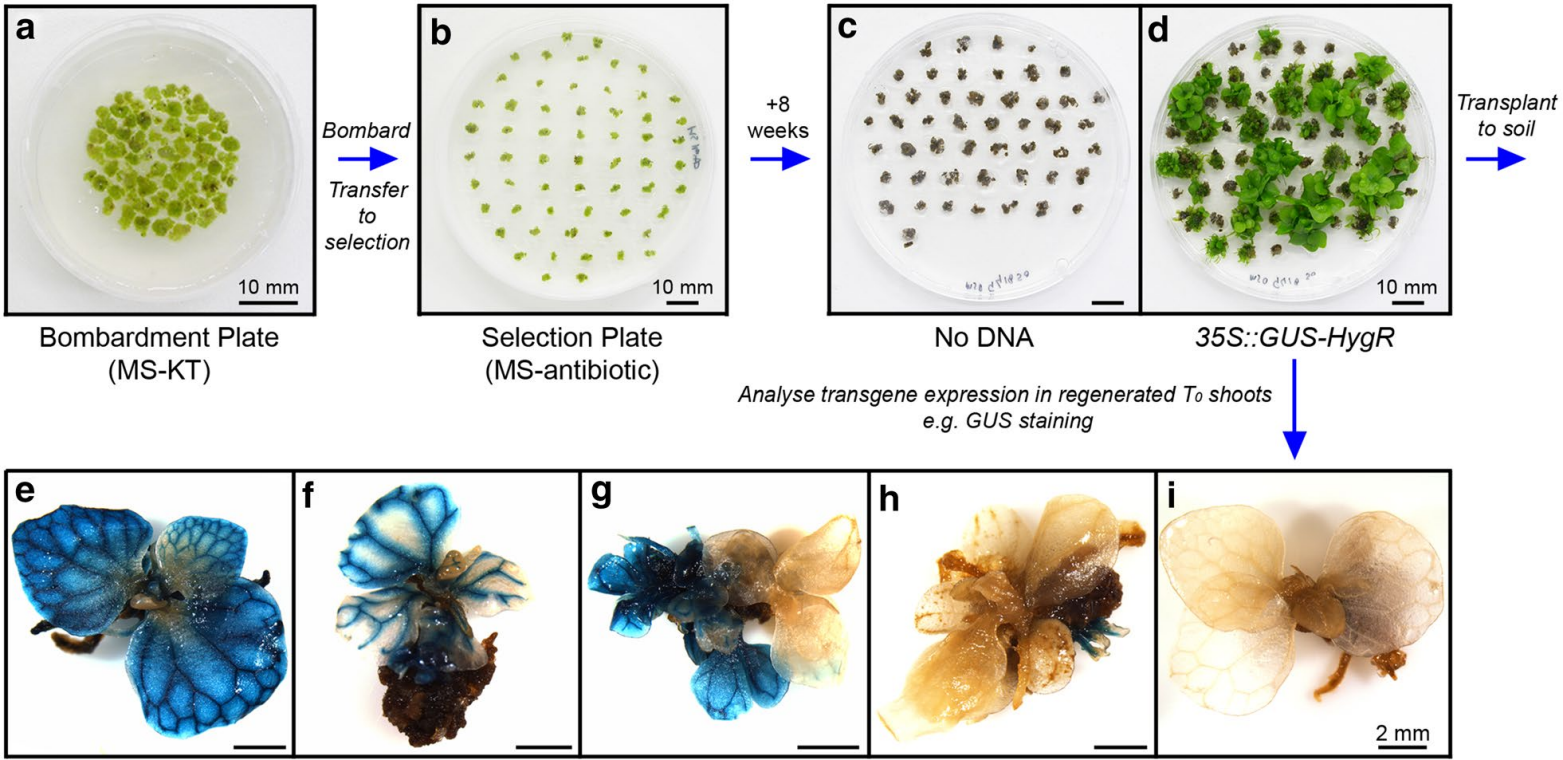
Callus bombardment and T_0_ sporophyte regeneration. Genetic transformation of *C. richardii* callus is through particle bombardment (**a**–**d**). Example shown is transformation of *35S::GUS*-*HygR*. Callus is assembled for bombardment on a 50 mm plate containing 1× MS 2% sucrose 0.7% agar media (pH 5.8) containing 5 μM KT (MS-KT), creating a dense 25–30 mm diameter target for bombardment. After bombardment callus is transferred onto MS-KT plates containing antibiotic (**b**) and selection is maintained for 8 weeks, transferring to fresh selective media every 2 weeks. KT treatment is removed after 2 weeks to allow tissue differentiation. Untransformed callus is killed and turns *dark brown* without further growth (**c**). Transformed callus cells survive and divide, regenerating into differentiated sporophytes (**d**) suitable for transplanting to soil. Multiple sporophytes can regenerate from the same piece of callus. Transgene expression within regenerated sporophytes is variable (**e**–**f**), with some demonstrating full transformation (**e**), others apparently chimeric expression (**f**) and some demonstrating no expression despite antibiotic resistance (**g**–**i**).
2. Prepare sterile tungsten microparticles and label with plasmid DNA for transformation [15] (see detailed protocol in Additional file 7). Sterilisation and labelling of microparticles can be conducted on the bench, outside of a laminar flow cabinet.
3. Bombard callus tissue with the DNA of interest under sterile conditions, following the set-up and operating protocols specified by the manufacturer of the bombardment system.
  *In our hands* Whilst successful transformation of callus occurred across the entire range of firing pressures tested [400–1,100 pounds per square inch (psi)] using the Bio-Rad PDS-1000/He™ system, bombardment at 600 psi resulted in the greatest degree of regeneration and confirmed transformation of T_0_ sporophytes (Additional file 6).
  *Note* It is strongly recommended that at least one ‘No DNA’ control (unlabelled microparticles) be bombarded at the start of each transformation session to screen for successful antibiotic selection and for the presence of cross-contamination from prior bombardments. It is recommended that multiple bombardments of the same plasmid be performed to ensure the creation of independent transgenic lines. If multiple plasmids are being bombarded within the same session, it is further recommended that plasmids carrying different antibiotic selection markers be bombarded to minimize the chances of cross-contamination, and that a further No DNA control be bombarded between bombardments of the different plasmids.
4. Re-seal bombardment plates with parafilm and incubate them under 28°C LD growth conditions for 1–3 days prior to transfer to selection media.
  *Note* Whilst acceptable frequencies of regeneration and transformation were achieved after only an overnight recovery post-bombardment before moving callus onto selection, the greatest proportion of fully-transgenic sporophytes suitable for transplant to soil were obtained after a 3 days recovery interval (Additional file 6).
5. Transfer callus onto MS-KT 90 mm selection plates containing antibiotic at the appropriate concentration (Table 1) and incubate plates under 28°C LD growth conditions. Individual callus pieces should be spaced separately (Figure 3b) to allow for ease of selection of independent regenerated shoots. Typically all callus tissue from one bombardment plate can fit onto a single 90 mm selection plate.
  *Important* Selection plates MUST be sealed with parafilm to retain high humidity, otherwise shoot regeneration will fail.
6. Maintain callus under continuous antibiotic selection for 8 weeks, transferring callus to fresh MS-antibiotic medium every 2 weeks. Regenerated sporophytes are typically large enough to transplant to soil by 8 weeks (20–30 mm rosette diameter, Figure 3d).
  *Note* After the first 2 weeks KT should not be included in the regeneration medium: continued application of KT is detrimental to the quality of regenerated shoots (Additional file 6). Callus tissue typically begins turning brown by 2 weeks after the start of selection, and is entirely brown by 4–6 weeks (Figure 3c). Sporophyte regeneration is typically visible from 4 weeks as small green nodes growing from otherwise brown/black callus.
  *Note* The continued growth of regenerating sporophytes can result in crowding. It is recommended that at 6 weeks after bombardment regenerating callus tissue from each bombardment be spaced over two selection plates instead of one. Frequently multiple sporophytes will regenerate from the same piece of callus. It is important for subsequent transgenic selection (step B7) that sporophyte clusters arising from the same callus not be broken up during selection.
  *Troubleshooting* If selection has been successful, the accompanying No DNA control plates should show no evidence of sporophyte regeneration (Figure 3c). If callus tissue remains substantially green after 4–6 weeks selection, including the No DNA control, antibiotic selection is failing. Transfer callus to media prepared with a fresh antibiotic stock. If sporophyte regeneration occurs on the No DNA control plate in addition to the test bombardments, contamination with DNA from this or previous bombardments has probably occurred. Clean all biolistic gun components thoroughly prior to any repeat bombardments—it is recommended that No DNA bombardments be tested first to confirm the absence of contamination. If sporophyte regeneration has failed on all plates, check that the resistance marker for the plasmid in question is functional. If sporophyte regeneration persistently fails, check that regeneration conditions are correct and ensure that plates are being sealed with parafilm.
7. Unseal tissue culture plates and transplant individual T_0_ sporophytes to soil using forceps (see detailed protocol in Additional file 4). Any T_0_ sporophytes not transplanted to soil can be analysed to assess transgene expression.
  *Important* Multiple independent transgenic lines should be established for each transgene to obtain a reliable and accurate record of transgene expression and function. Sporophytes obtained from separate bombardments represent independent transformation events, but individuals from the same bombardment could be clones of the same initial genomic integration [9]. This probability increases in sporophytes regenerating from the same piece of callus. Proof-of-principle experiments found that a single regenerated population of T_0_ sporophytes contains a range of transgene expression patterns [9] (Figure 3e–i). Not all individuals regenerating from the same callus express the desired transgene when tested (Figure 3g–i), and transgene expression was not found to equate with regenerated sporophyte size. To maximise the number of independent lines obtained when transplanting sporophytes to soil, it is therefore recommended that only a single individual be transplanted from any particular callus cluster. In our experience, a relatively reliable indicator of genuine antibiotic resistance in a regenerated shoot is the growth of roots into the selection media.
8. Grow T_0_ sporophytes to maturity on soil. Regenerated sporophytes take 2–3 months to begin producing reproductive fronds (Figure 1g) containing T_1_ spores.
9. Harvest mature reproductive fronds from individual T_0_ sporophytes and collect T_1_ spores (see detailed protocol in Additional file 4). Take care to avoid cross-contamination between individuals when harvesting.
  *Note* In our experience *C. richardii* spores retain dormancy for at least 1 month after harvesting, with very poor spore germination observed if fresh spores are imbibed during this time. An after-ripening period of at least 2 months after harvesting is therefore recommended to make screening of T_1_ lines more reliable.

#### C. Selection of T_1_ lines

Because of the apparently chimeric nature of many regenerated T_0_ sporophytes, not all T_1_ spores harvested carry the desired transgene. As such, each T_1_ line must be screened to identify transgenic individuals, the frequency of which can vary by a wide degree [9]. Furthermore, in proof-of-principle experiments antibiotic selection rendered even resistant T_1_ gametophytes sterile [9], necessitating the induction of T_1_ sporophytes in the absence of selection at the gametophyte stage, with subsequent identification of transgenic sporophytes through separate antibiotic resistance screening. Although some examples of transgenic sporophytes forming directly from transgenic gametophytes have since been observed (Additional file 8), this is not universal and potentially dependent on the selection marker used. The protocol described below assumes separate induction and screening of sporophyte populations for antibiotic resistance.

*T*_*1*_* gametophyte screening*

**Figure 4:**
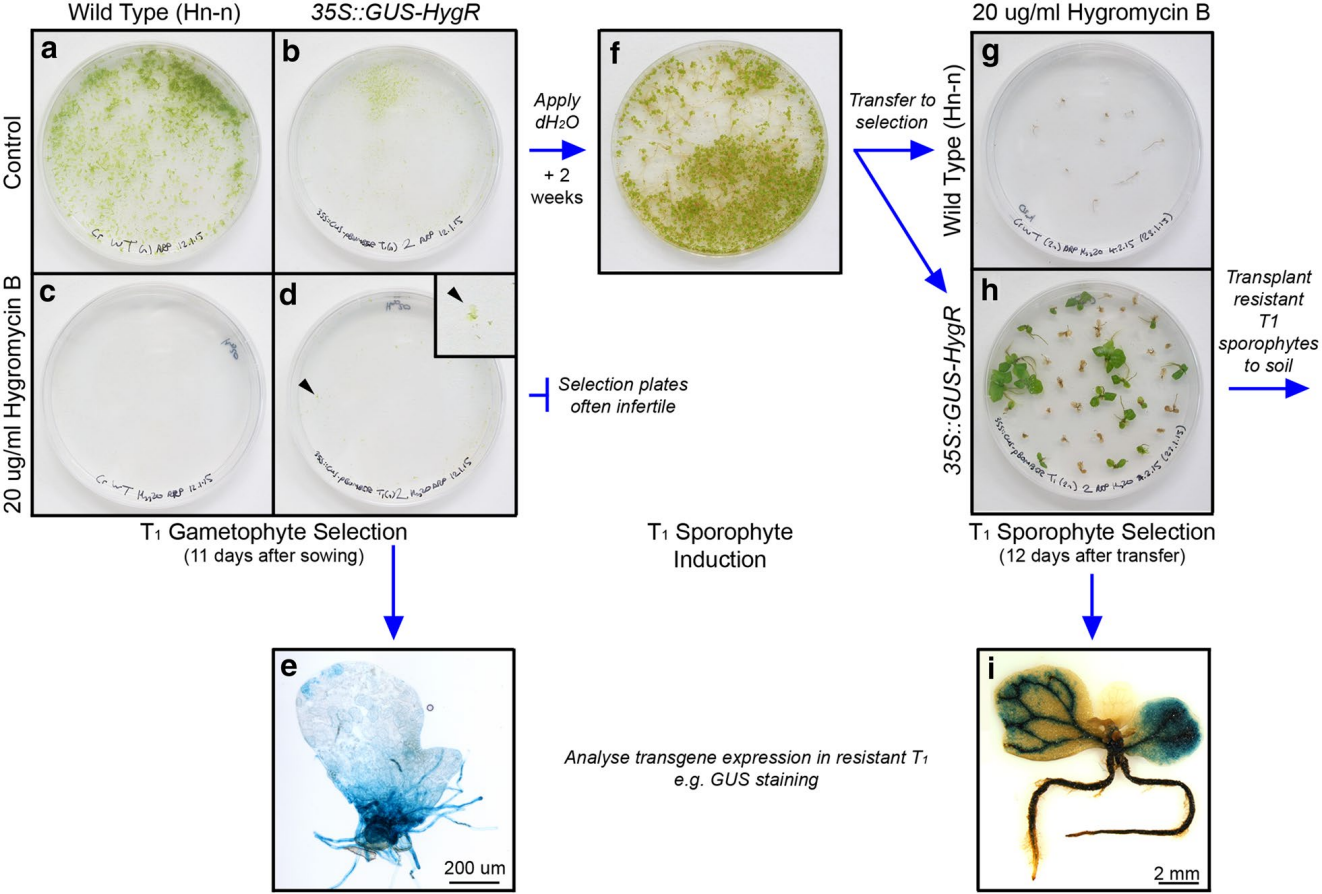
Selection of T_1_ transgenic lines. Transgenic T_1_ individuals are selected for inherited antibiotic resistance separately at the gametophyte (**a**–**e**) and sporophyte (**f**–**i**) stages. Example shown is *35S::GUS*-*HygR*. **a**–**e** Gametophyte selection. Spores are sown on two C-fern 1% agar (pH 6.0) plates: one containing antibiotic (selective plate), the other not (control plate). By 11 days after sowing, WT (**a**) and T_1_ (**b**) gametophyte populations have developed on the control plates. Under antibiotic selection, WT gametophytes have not developed (**c**) but some resistant T_1_ gametophytes have developed successfully (**d**). *Inset* shows resistant gametophyte developing on the plate (*arrowhead*). Resistant T_1_ gametophytes can then be analysed for transgene expression in addition to antibiotic resistance (**e**). **f**–**i** Sporophyte selection. T_1_ sporophytes are induced from the control gametophyte plate by repeated application of sterile water. Sporophytes typically become visible on the plate within 2 weeks (**f**). Individual sporophytes are then transferred to C-fern 1% agar (pH 6.0) plates containing antibiotic to screen for antibiotic resistance. Selection typically requires 7–14 days. Antibiotic selection kills WT (**g**) and non-transgenic T_1_ sporophytes (**h**), whilst transgenic T_1_ sporophytes within the population survive and continue to grow (**h**). After antibiotic selection resistant T_1_ sporophytes can be transplanted to soil, or analysed for transgene expression in addition to antibiotic resistance (**i**).

1. Sterilise an aliquot of spores for each T_1_ line to be screened (see detailed protocol in Additional file 4) including an aliquot of untransformed wild type (WT) spores as a control. Incubate at room temperature in darkness for 3–5 days.
2. Sow each spore line over two C-fern medium 90 mm diameter tissue culture plates, one without antibiotic selection and one containing antibiotic at the appropriate concentration (Table 1). Culture gametophytes under sterile conditions until 9–11 days old (see detailed protocol in Additional file 4).
  *Note* If further analysis of resistant T_1_ gametophytes is required, e.g. GUS staining or fluorescence microscopy, their removal from the plate can be facilitated by placing a sterile cellophane disc over the medium prior to spore sowing (see detailed protocol in Additional file 4). It is recommended that cellophane discs not be used for control plates if sporophytes are to be induced and grown from them, as they interfere with root penetration into the growth medium.
3. Screen T_1_ lines visually for antibiotic resistance. Control plates should carry developing gametophytes for all lines (Figure 4a, b). Antibiotic selection should kill all germinating untransformed spores [9], resulting in no visible gametophyte growth on the WT antibiotic selection plate (Figure 4c). Transgenic T_1_ gametophytes with antibiotic resistance should develop and be visible in the presence of antibiotic (Figure 4d).
  *Note* After screening, resistant gametophytes can then either be analysed further for transgene expression (Figure 4e) or attempts can be made to induce sporophytes by adding sterile water to the plate.
  *Note* Growth of gametophytes under antibiotic selection can be slower than under control conditions, and their precise developmental stage will need to be confirmed visually prior to screening.
  *Troubleshooting* If gametophytes are developing on the WT antibiotic plate, selection has not worked effectively. The antibiotic stock may have become degraded, or contamination of the WT spore stock with transgenic spores might have occurred. Repeating the screen using a fresh antibiotic stock should determine this. If no gametophytes develop for a T_1_ line on either control or selective media, spore sterilisation might have been too severe or the spore stock may not be viable. Repeat the selection screen, sterilising spore stocks for a shorter period (5–10 min).

*T*_*1*_* sporophyte screening*

4. Induce sporophytes from the control plate of each line by adding sterile water (see step A2).
  *Note* T_1_ sporophytes induced without selection from a mixed population of transgenic and non-transgenic gametophytes may or may not be transgenic, and so must also be screened for antibiotic resistance.
5. When sporophytes are visible on the fertilized gametophyte plates (Figure 4f) transfer individual sporophytes to 90 mm C-fern plates containing antibiotic at the appropriate concentration (Table 1). After transferring, add 2 ml sterile water drop-wise to the plate with a sterile Pasteur pipette to increase humidity. Seal each plate with micropore tape and incubate under 28°C LD growth conditions. If plates become dry during incubation, repeat the addition of 2 ml sterile water every 4–5 days.
  *Note* Approximately 25 sporophytes can be grown on a single selective plate. It is recommended that WT sporophytes be included in each selective screen for comparison against T_1_ sporophytes, and that some WT sporophytes be grown on media without antibiotic selection to provide comparisons against resistant T_1_ lines at future growth stages.
6. Screen sporophytes visually for antibiotic resistance. Sporophytes lacking antibiotic resistance typically die 7–14 days after the start of selection (Figure 4g), whilst resistant sporophytes continue to grow (Figure 4h).
7. Transplant resistant sporophytes to soil for further growth (see Additional file 4).
  *Note* It is recommended that at least 4 resistant individual sporophytes from each T_1_ line be grown on soil to provide an assessment of the variability of transgene expression and genomic T-DNA insertions within that particular line. Any resistant T_1_ sporophytes not transplanted to soil can be analysed for transgene expression at this stage (Figure 4i).

## Comments

As a transformation technique, particle bombardment has a number of disadvantages compared to techniques such as *Agrobacterium*-mediated transformation. Most notably, transgenics regenerated from particle bombardment typically contain a greater number of copies of the inserted transgene than those transformed by *Agrobacterium* [16]. Furthermore, physical shearing of T-DNA on impact with the plant cells results in the incorporation of fragmentary T-DNA, and the separation of the transgene cassette of interest from the antibiotic selection marker. All of these make the interpretation of any transgenic phenotypes and expression patterns more complex. As a consequence, it is important that any *C. richardii* transgenic lines produced through particle bombardment be evaluated thoroughly for copy number and the presence of full-length T-DNA. This can be achieved in the T_1_ generation through genotyping PCR and Southern analysis [8, 9]. If lines carrying single copy insertions are not identified in the T_1_ generation, T-DNA copy number can be reduced in subsequent generations by back-crossing to WT: one of the advantages of *C. richardii* as an experimental system is that sexual reproduction occurs under tissue culture conditions and can thus be tightly controlled.

Particle bombardment is applicable to a broader range of plant species than *Agrobacterium*-mediated transformation, as many are not naturally susceptible to infection [17]. In the case of *C. richardii*, *Agrobacterium* infection of gametophytes resulted in transformation at low efficiencies with practical difficulties associated with selection of transgenics [8], and attempts to transform sporophytic callus tissue using *Agrobacterium* were unsuccessful [9].

## Conclusions

Genetic transformation of *C. richardii* through particle bombardment was found to be robust under a broad range of bombardment and recovery conditions, and requires relatively little specialist knowledge to implement beyond familiarity with tissue culture. As such, this protocol represents a practicable method of generating transgenic lines in sufficient quantity for reliable evaluation of transgene function and establishing it in new labs should be relatively simple. The successful expansion of the selection toolkit to include a second antibiotic for resistance screening (G-418) increases the scope of transformations possible using this technique and offers the prospect of more complex genetic analysis, for example the creation of lines carrying more than one transgene. The introduction of a robust and practicable transformation technique represents a very important milestone in the field of fern biology, and its successful implementation in *C. richardii* paves the way for adoption of this species as the first fern genetic model.

## Additional files

Additional file 1: Media for *C. richardii* tissue culture. Protocols for the preparation of C-fern and MS agar media used during *C. richardii* transformation. Additional file 2: G-418 selection conditions for *C. richardii*. Kill curves demonstrating the necessary concentration of G-418 antibiotic needed for selection of *C. richardii* callus, gametophytes and sporophytes. Additional file 3: Evaluation of antibiotic selection markers. Design of constructs used to test promoter function driving HygR and NPTII resistance markers, and the mean regeneration frequencies achieved. Additional file 4: Culturing protocols for *C. richardii*. Protocols for the preparation and growth of *C. richardii* in sterile tissue culture and on soil. Additional file 5: Evaluation of callogenic hormone treatments. Comparison of two hormone treatments (BAP + IBA vs. BAP only) on callus maintenance and callus transformation efficiency. Additional file 6: Optimisation of bombardment conditions. Results of test *35S::GUS*-*HygR* transformation efficiencies under different bombardment conditions: KT treatment during regeneration, He_2_ firing pressure and recovery interval length. Additional file 7: Preparation of microparticles for bombardment. Protocols for the sterilisation and DNA-labelling of microparticles for bombardment. Additional file 8: Direct induction of transgenic sporophytes from gametophytes under antibiotic selection. An example of the successful generation of T_1_ sporophytes from gametophytes under antibiotic selection and subsequent confirmation of their transgenic status.

## Abbreviations

BAP: 6-benzylaminopurine
bp: base pairs
CK: cytokinin
EDTA: ethylenediaminetetraacetic acid
EtOH: ethanol
GUS: β-glucuronidase
He_2_: helium (gas)
HygR: hygromycin B phosphotransferase
KT: kinetin
LD: long day
MS: Murashige and Skoog
nos: nopaline synthase
NPT: neomycin phosphotransferase
psi: pounds per square inch
rpm: revolutions per minute
S.E.: standard error
T-DNA: transgene DNA
WT: wild type
