# Supplementary material for: Protocol: genetic transformation of the fern Ceratopteris richardii through microparticle bombardment
Source: Plant Methods. 2015 Jul 3;11:37. doi: 10.1186/s13007-015-0080-8 (PMC4490597; doi:10.1186/s13007-015-0080-8)
Supplement: Additional file 1: — Media for C. richardii tissue culture. Protocols for the preparation of C-fern and MS agar media used during C. richardii transformation. [file 13007_2015_80_MOESM1_ESM.pdf]

Additional File 1: Media for *C. richardii* tissue culture.

C-fern medium

A. Preparation of stock solutions

1. C-fern medium is prepared most easily from stock solutions. Prepare 1 L stock solutions in advance using deionised water, as follows:

**Stock Solution ID**	**Stock Solution Concentration (Nx)**	**Nutrient**	**Stock Concentration (g/L)**
Macronutrients	10x	NH_4NO_3	1.2500
		KH_2PO_4	5.0000
		$MgSO_4.7H_2O$	1.2000
		$CaCl_2.2H_2O$	0.2600
Chelated Iron (Fe-EDTA)	100x	$FeSO_4.7H_2O$	2.7800
		Disodium EDTA.$2H_2O$	3.7300
Micronutrients	200x	$MnSO_4.1H_2O$	0.0500
		$CuSO_4.5H_2O$	0.0740
		$ZnSO_4.7H_2O$	0.1040
		H_3BO_3	0.3720
		$(NH_4)_6Mo_6O_{24}.4H_2O$	0.0074

2. Autoclave macronutrients and micronutrients stock solutions after preparation (20 minutes, 121°C, 15 psi) to sterilise.

3. To prepare the Fe-EDTA stock solution, dissolve the $FeSO_4$ and EDTA separately, each in ≈ 450 ml deionised water. Adjust the pH of EDTA to ≥ 7.0 to dissolve the EDTA powder. After the powder is dissolved, adjust the final EDTA pH to 7.0. Heat the EDTA solution to boiling and then add it to the $FeSO_4$ solution. Boil the combined solution for one hour (covered), then allow to cool completely. Adjust the volume to 1 L using deionised water.

4. Store stock solutions at 4°C.

B. Preparation of 1x C-fern 1% agar medium (pH 6.0).

1. Combine 100 ml of the macronutrients solution, 10 ml Fe-EDTA and 5 ml micronutrients stock solutions in ≈ 750 ml deionised water.

2. Adjust the pH to 6.0 using 1 M NaOH or 1 M KOH solution. Make up the final volume to 1 L using deionised water.

3. Divide the C-fern solution into 500 ml autoclavable bottles, add agar powder to a final concentration of 1% (w/v) and autoclave (20 minutes, 121°C, 15 psi) to sterilise. Autoclaved medium can be used directly or stored at 4°C.

4. Prepare sterile tissue culture plates (90 mm diameter x 15 mm depth) in a sterile laminar flow hood. Each plate holds 25 ml agar medium. Melt the medium in a microwave, allow to cool to 45°C, add antibiotic stocks of the appropriate concentration if necessary, and then pour into the plates. Allow the plates to cool and set. Plates can be used immediately or stored at 4°C.

MS medium

C. Preparation of 1x MS 2% sucrose 0.7% agar medium (pH 5.8).

1. For 1 L of media add the following to 850 ml of deionised water:

   - 4.16 g Murashige and Skoog (MS) medium (including vitamins).
   - 0.5 g MES free acid.
   - 20 g sucrose.

2. Once dissolved, adjust the pH to 5.8 using 1 M NaOH or 1 M KOH solution. Make up the final volume to 1 L using deionised water.

3. Divide the MS-sucrose solution into 500 ml autoclavable bottles, add agar powder to a final concentration of 0.7% (w/v) and autoclave (20 minutes, 121°C, 15 psi) to sterilise. Autoclaved medium can be used directly or stored at 4°C.

4. Prepare sterile tissue culture plates (90 mm diameter x 15 mm depth or 50 mm diameter x 20 mm depth, as required) in a sterile laminar flow hood. Each plate holds 25 ml (90 mm diameter) or 15 ml (50 mm diameter) of agar medium, respectively. Melt the medium in a microwave, allow to cool to 45°C, add antibiotic or hormone stocks of the appropriate concentration if necessary and then pour into the plates. Allow the plates to cool and set. Plates can be used immediately or stored at 4°C.
