# Supplementary material for: Protocol: genetic transformation of the fern Ceratopteris richardii through microparticle bombardment
Source: Plant Methods. 2015 Jul 3;11:37. doi: 10.1186/s13007-015-0080-8 (PMC4490597; doi:10.1186/s13007-015-0080-8)
Supplement: Additional file 2: — G-418 selection conditions for C. richardii. Kill curves demonstrating the necessary concentration of G-418 antibiotic needed for selection of C. richardii callus, gametophytes and sporophytes. [file 13007_2015_80_MOESM2_ESM.pdf]

Additional File 2: G-418 selection conditions for *C. richardii*.

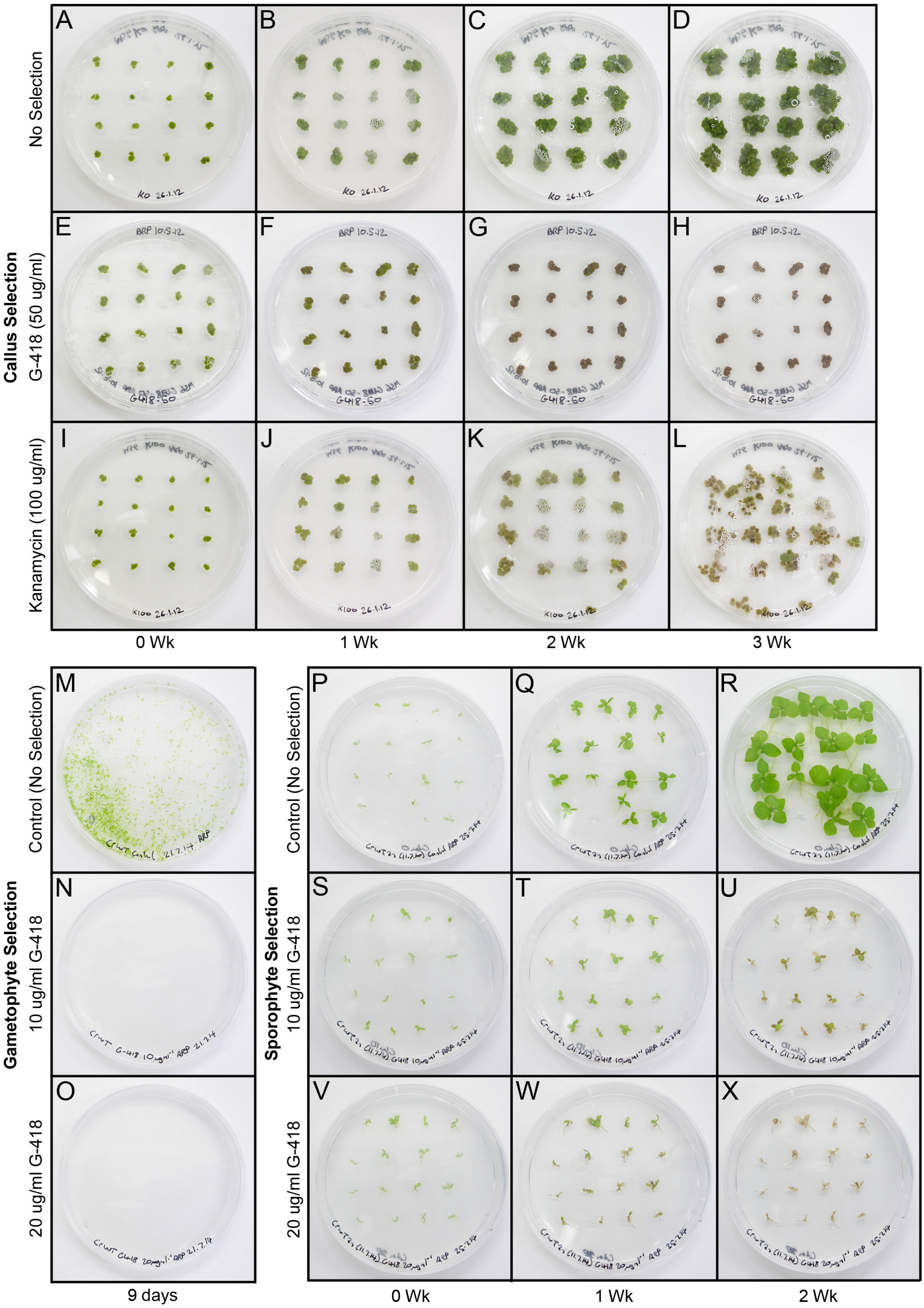

A-L: Selection conditions for regenerating *C. richardii* callus on MS medium. Callus tissue was transferred to MS-sucrose medium and incubated under 28°C LD conditions for three weeks. Growth of callus tissue was recorded photographically each week. In the absence of antibiotic (A-D), callus grew continuously in tissue culture. A concentration of 50 μg/ml of the kanamycin analogue G-418 was sufficient to kill callus within two weeks (E-H): callus tissue began turning brown after one week (F) and growth had ceased by two weeks after transfer onto antibiotic (G). In contrast, kanamycin monosulphate was not effective at completely killing callus, even at concentrations of 100 μg/ml (I-L): green callus persisted up to three weeks after transfer (L).

M-O: Selection conditions for *C. richardii* gametophytes on C-fern medium. In the absence of G-418 (M), wild-type spores germinate and by nine days after sowing gametophytes are clearly distinguishable on the plate surface. When germinated on medium containing G-418 (N, O) wild-type spores are killed and gametophytes do not develop. A concentration of 10 μg/ml G-418 is sufficient to kill untransformed *C. richardii* gametophytes.

P-X: Selection conditions for *C. richardii* sporophytes on C-fern medium. Sporophytes transferred to C-fern media without G-418 (P-R) remain green and increase in size over two weeks as they continue to produce new fronds. Growth over two weeks is inhibited by the addition of 10 μg/ml G-418 (S-U), but some sporophytes remain green after this time (U). A concentration of 20 μg/ml G-418 is sufficient to kill untransformed sporophytes within two weeks (V-X).
