# Supplementary material for: Protocol: genetic transformation of the fern Ceratopteris richardii through microparticle bombardment
Source: Plant Methods. 2015 Jul 3;11:37. doi: 10.1186/s13007-015-0080-8 (PMC4490597; doi:10.1186/s13007-015-0080-8)
Supplement: Additional file 3: — Evaluation of antibiotic selection markers. Design of constructs used to test promoter function driving HygR and NPTII resistance markers, and the mean regeneration frequencies achieved. [file 13007_2015_80_MOESM3_ESM.pdf]

Additional File 3: Evaluation of antibiotic selection markers.

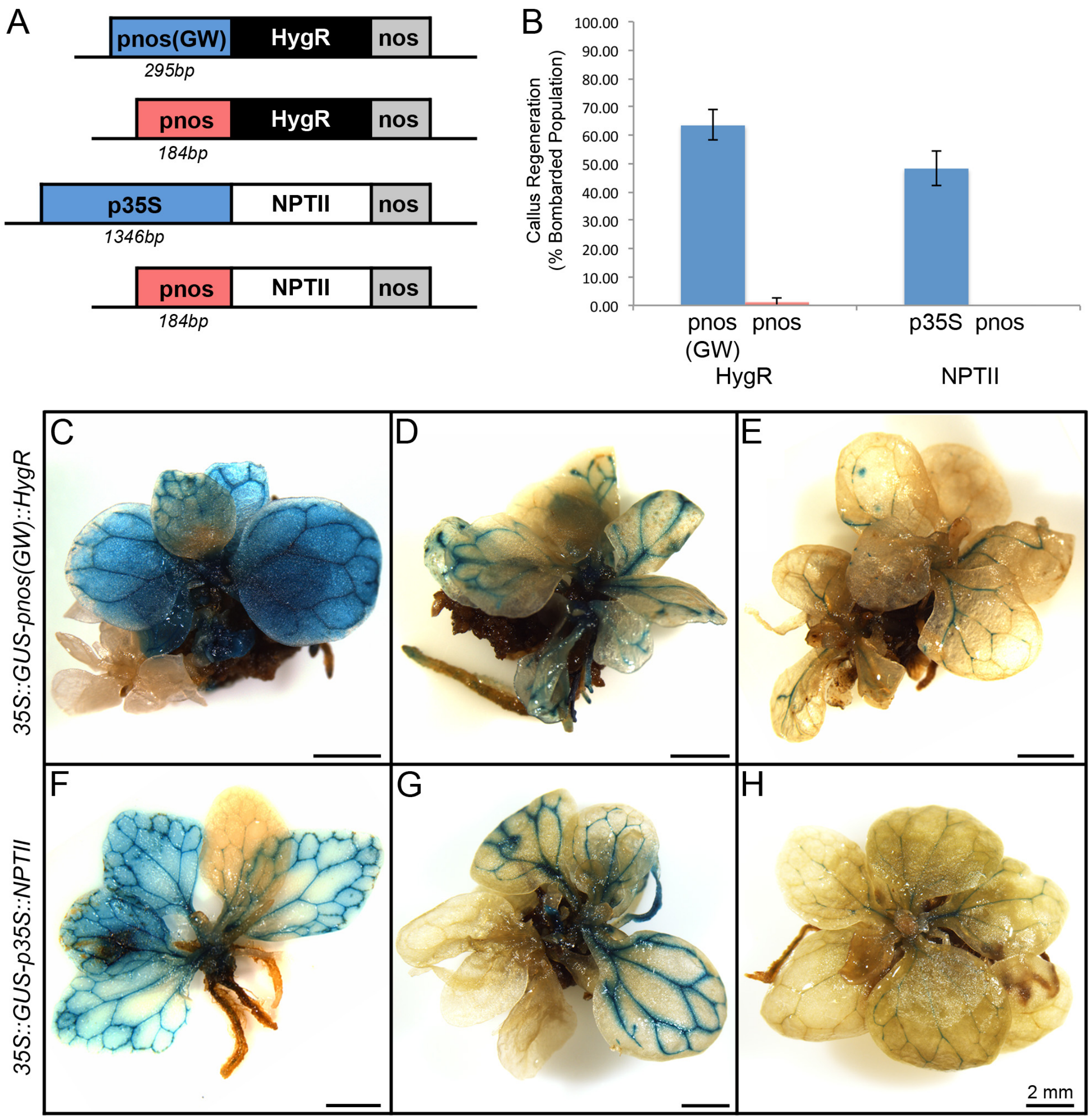

Four resistance cassettes (A) were bombarded separately into *C. richardii* callus, each linked to a *35S::GUS* expression cassette. The HygR resistance marker was driven separately by two variants of the nos promoter, one used in Gateway vectors (pnosGW) [12] and the second in the pART27 plant transformation vector (pnos) [13]. The viral 35S promoter has previously been shown to drive sufficient HygR expression for use in antibiotic resistance screening [9]. To test functionality of G-418 selection, the NPTII resistance marker was driven separately by the 35S promoter

(p35S) and pnos. Three replicate bombardments were performed using each selection marker, and all constructs were bombarded and regenerated simultaneously. Successful regeneration was achieved using pnos(GW) driving HygR and 35S driving NPTII (B), but pnos failed to regenerate transgenics when driving either HygR or NPTII. The regeneration of genuine transgenics was confirmed through GUS staining assay (C-H). A similar range of GUS expression patterns were observed in regenerated T_0 sporophytes using either the HygR (C-E) or the NPTII selection tags (F-H).
