# Supplementary material for: Protocol: genetic transformation of the fern Ceratopteris richardii through microparticle bombardment
Source: Plant Methods. 2015 Jul 3;11:37. doi: 10.1186/s13007-015-0080-8 (PMC4490597; doi:10.1186/s13007-015-0080-8)
Supplement: Additional file 4: — Culturing protocols for C. richardii. Protocols for the preparation and growth of C. richardii in sterile tissue culture and on soil. [file 13007_2015_80_MOESM4_ESM.pdf]

Additional File 4: Culturing protocols for *C. richardii*.

Both the gametophyte and sporophyte stages of *C. richardii* can be grown in sterile culture, although for greatest productivity the sporophyte is best grown on soil beyond approximately one month after induction. For convenience, the *C. richardii* lifecycle is typically considered as going from spore-to-spore (Fig. 1). Spores can be stored dry at room temperature in envelopes or plastic tubes and remain viable for several years.

A. GAMETOPHYTE STERILE CULTURE

1. Sterilise an aliquot of spores (≤ 20 mg) in a 2 ml microcentrifuge tube by incubating in 1 ml diluted bleach solution (2% chlorine, 0.1% Tween-20 (v/v)), for 10-15 minutes at room temperature. Spores typically become lighter in colour during incubation.

NOTE: If a spore stock is contaminated e.g. with frond tissue, this is best removed prior to sterilising using the following method: add 1 ml water to the dry spores, vortex briefly to suspend, allow the contaminating tissue to float to the surface (a few seconds), then pipette the spores in solution to a fresh tube.

NOTE: Solutions are removed from spores most effectively with a sterile Pasteur pipette, using the technique described in [11], as follows. Evacuate air from the pipette by squeezing and press the pipette tip against the bottom of the tube, then gently withdraw the solution whilst maintaining downward pressure. As the solution is removed spores will collect against the outer edge of the pipette tip, becoming visible as a brown pellet.

NOTE: All subsequent steps are conducted under sterile conditions in a laminar flow cabinet.

2. Remove the bleach solution and rinse the spores in four changes of 1 ml sterile water. Imbibe the spores in 1 ml sterile water, seal the tube in foil and incubate them at room temperature.

NOTE: To synchronize gametophyte development, imbibed spores should be incubated in the dark at room temperature for at least 48 hours prior to sowing [11]. In our experience spore development will remain arrested prior to germination in the dark for up to seven days.

3. Prepare C-fern 90 mm diameter tissue culture plates (see Additional File 1). Additional hormone or antibiotic treatments can be added to the molten media prior to pouring once the media is sufficiently cool (45°C). A 20 mg aliquot of imbibed spores is sufficient to sow up to eight gametophyte plates.

OPTIONAL: If gametophytes are going to be removed from the plate for analysis during development, a sterile cellophane disc (90 mm diameter) can be laid over the surface of the medium to make gametophyte recovery easier without significantly affecting gametophyte development or response to hormone or antibiotic treatments.

a. Place single cellophane discs between filter papers in an alternating stack, depending on the numbers required, beginning and ending with filter paper.

b. Place the stack inside an autoclavable tissue-culture plate or glass petri dish and lid, then seal with aluminium foil inside a waterproof container (e.g. a Gilson pipette tip-box) and autoclave (20 minutes, 121°C, 15 psi) to sterilise.

c. In a sterile laminar flow hood, prepare an empty 90 mm diameter plate with sterile water to act as a waterbath. Unbox, unwrap and open the dish of sterile cellophane, taking care not to disturb the stack.

d. Using two pairs of sterile forceps, remove a cellophane disc and submerge it in the waterbath until fully wetted, then remove from the water and lay it over the agar surface of a plate. Remove any air-bubbles from beneath the cellophane.

4. Sow spores in solution dropwise onto the plate surface.

NOTE: Depending on plate numbers, it is recommended that a maximum of 0.5 ml be sown per plate to avoid overcrowding. To spread spores evenly across the media, add 2 ml sterile water to each plate, then swirl gently until entire surface is covered.

NOTE: In our experience *C. richardii* spores germinate poorly if too deeply submerged. Leave plates open for excess water to evaporate before sealing. Ideally some surface water should still be visible, but with spores emerging above the surface of the water.

5. Seal plates with micropore tape and incubate at 28°C under long day (LD) conditions.

NOTE: Controlled humidity is not essential at this stage, but optimum growth is achieved if plate humidity is kept relatively high. Add 1.5 ml sterile water if plate surface looks dry, in our experience typically at three, five and seven days after sowing in the absence of controlled humidity. Keeping plates relatively dry after seven days allows a degree of control over the timing of sporophyte induction (see step 6).

TROUBLESHOOTING: If plates show signs of bacterial or fungal contamination, a longer sterilisation period might be required for that particular batch of spores, media stocks may not have been sterilised correctly or contamination is being introduced during tissue culture. To determine the cause, pour an additional plate from the same media stock as a control and incubate without spores alongside the gametophyte population.

B. SPOROPHYTE STERILE CULTURE

6. Add 4-5 ml sterile water to each gametophyte plate to fertilize the gametophyte population and induce sporophyte development. Swirl the plate gently to distribute the water evenly, then reseal and incubate at 28°C under LD conditions. To maximize fertilization, repeat this step after 3-4 days. Subsequently, maintain high humidity by adding 2 ml sterile water to each plate every 4-5 days.

NOTE: If fertilization is successful, young sporophytes become visible typically seven days after induction, visible on the gametophyte thallus as a darker green frond and developing root (Fig. 1E). Sporophytes can continue growing on the gametophyte plate *en masse* for up to two weeks after fertilization.

7. Two weeks after induction, transfer individual sporophytes to fresh C-fern plates using sterile forceps to continue their growth. Add 2 ml of sterile water to each plate to increase humidity, seal with micropore tape and incubate at 28°C under LD conditions.

NOTE: Approximately 25 sporophytes can be grown on a single 90 mm plate. Once transferred, maintain high humidity by adding 2 ml sterile water to each plate every 4-5 days. Sporophytes can continue to grow on these plates for 2-3 weeks (4-5 weeks after sporophyte initiation) before further transplanting is necessary.

8. Transfer one month-old sporophytes to soil (see section C below) or to prolonged sterile culture in Magenta pots containing 100 ml C-fern medium.

C. SPOROPHYTE GROWTH ON SOIL

Sporophytes can be transplanted to soil one month after induction (2-3 cm in size). Sporophytes can be grown to maturity in a 24-celled (5 x 5 cm cells) soil tray containing potting compost (e.g. Levington F3).

1. Prepare a P24 soil tray with compost, removing one corner well to increase ease of watering. Once the tray is filled, soak the soil well with water and fill the tray reservoir before transplant.

2. Transplant one month-old sporophytes to soil using watchmakers' forceps. Remove excess agar media from developing roots, dib a small hole in the centre of the soil cell using the forceps and press the sporophyte roots into it. Firm the soil around the sporophyte shoot.

3. Cover the soil tray with a transparent plastic propagator lid to maintain high humidity and transfer it to a controlled growth environment. Sporophytes should remain covered until they are successfully established and producing new fronds. The soil should be kept well-watered. Sporophytes typically take 3-4 months to reach maturity i.e. produce fully reproductive fronds (Fig. 1G).

IMPORTANT: *C. richardii* sporophytes are very sensitive to drying out, even when relatively mature. Sporophytes should be grown in controlled conditions of constant high humidity (80-85%) to maintain healthy development, at a constant temperature of 28°C. We find that *C. richardii* sporophytes develop well under an LD light cycle (16 hours light/8 hours dark) at a photon flux density of 120-150 $\mu mol/m^2/s$.

NOTE: Due to continued growth at high humidity, *C. richardii* sporophytes can be susceptible to attack by mildew/fungal pathogens. It is recommended that sporophyte trays be checked regularly, particularly if they become crowded. If contaminated fronds are discovered, these should be removed with scissors, cleaning with EtOH

between each to minimise cross-contamination. Once established, healthy growth of sporophytes is encouraged by regular removal of old vegetative fronds to clear space and increase light levels at the sporophyte apex.

4. Cut mature reproductive frond tissues from the sporophyte using scissors and forceps and store in glassine bags at room temperature (20-25°C) to dry out (2-4 weeks).

NOTE: Fronds containing sporangia can be harvested once the sporangia inside are visibly dark brown through the surrounding frond tissue. Reproductive fronds can take 3-4 weeks to mature after emerging from the apex.

5. Decant spores for long-term storage at room temperature, either in paper envelopes or plastic tubes. Spores can be released from dried frond tissues by applying gentle pressure and agitation, although care should be taken not to crush the tissue excessively.

NOTE: In our experience the simplest method of spore decanting is to cut the corner from the glassine bag and pour the released spores into a labelled microcentrifuge tube.

NOTE: *C. richardii* spores retain dormancy for at least one month after harvesting, with very poor spore germination observed if fresh spores are imbibed during this time. An after-ripening period of at least two months after harvesting is therefore recommended.
