# Supplementary material for: Protocol: genetic transformation of the fern Ceratopteris richardii through microparticle bombardment
Source: Plant Methods. 2015 Jul 3;11:37. doi: 10.1186/s13007-015-0080-8 (PMC4490597; doi:10.1186/s13007-015-0080-8)
Supplement: Additional file 5: — Evaluation of callogenic hormone treatments. Comparison of two hormone treatments (BAP + IBA vs. BAP only) on callus maintenance and callus transformation efficiency. [file 13007_2015_80_MOESM5_ESM.pdf]

Additional File 5: Evaluation of callogenic hormone treatments.

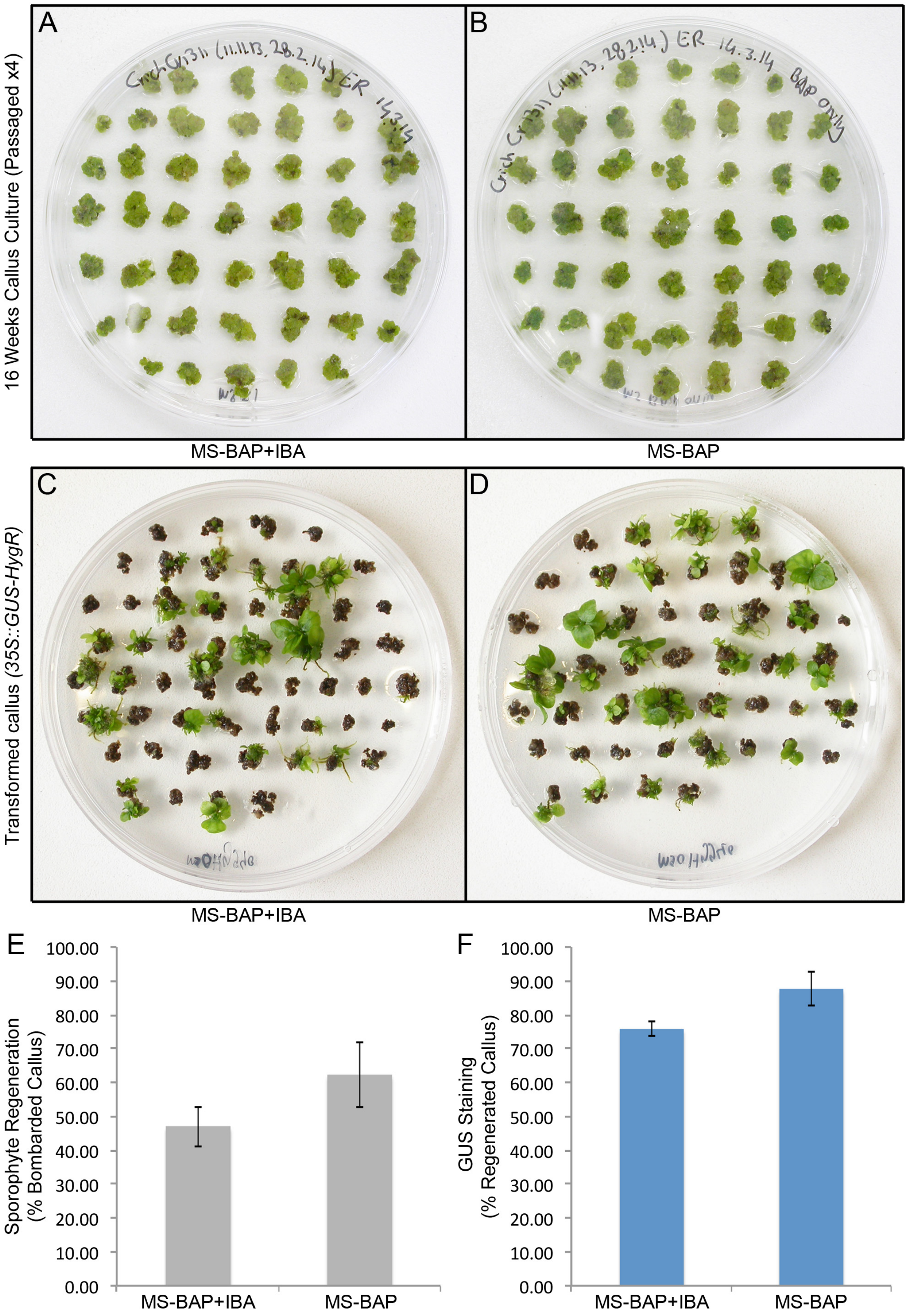

Original proof-of-principle bombardments were performed using callus induced and maintained on MS media containing 5 μM BAP and 5 μM indole-3-butyric acid (IBA) (MS-BAP+IBA) [9]. It was found that callus tissue can be induced by BAP treatment in the absence of IBA (MS-BAP; Fig. 2B). A comparative study induced and maintained callus separately on MS-BAP+IBA (A) and MS-BAP (B). After 16 weeks, callus stocks remained undifferentiated on MS-BAP. Side-by-side test bombardments were performed using a *35S::GUS-HygR* plasmid to test competence of the two callus stocks for transformation. Both callus stocks successfully regenerated transgenic T_0 sporophytes (C, D). The mean of three test bombardments ±S.E. show the effects of altering callus induction and maintenance conditions on the regeneration frequency of T_0 sporophytes as a percentage of callus bombarded (E) and the percentage of those regenerated sporophytes expressing GUS (F).
