# Supplementary material for: Protocol: genetic transformation of the fern Ceratopteris richardii through microparticle bombardment
Source: Plant Methods. 2015 Jul 3;11:37. doi: 10.1186/s13007-015-0080-8 (PMC4490597; doi:10.1186/s13007-015-0080-8)
Supplement: Additional file 6: — Optimisation of bombardment conditions. Results of test 35S::GUS-HygR transformation efficiencies under different bombardment conditions: KT treatment during regeneration, He2 firing pressure and recovery interval length. [file 13007_2015_80_MOESM6_ESM.pdf]

Additional File 6: Optimisation of bombardment conditions.

All optimisation experiments were conducted using a *35S::GUS* expression cassette linked to pnos(GW)-HygR (Additional File 3). Regeneration and transgenic status of all sporophytes was scored after eight weeks regeneration on 40 µg/ml hygromycin B. Three replicate bombardments of each treatment were performed. Values shown are the mean of each treatment ± S.E.

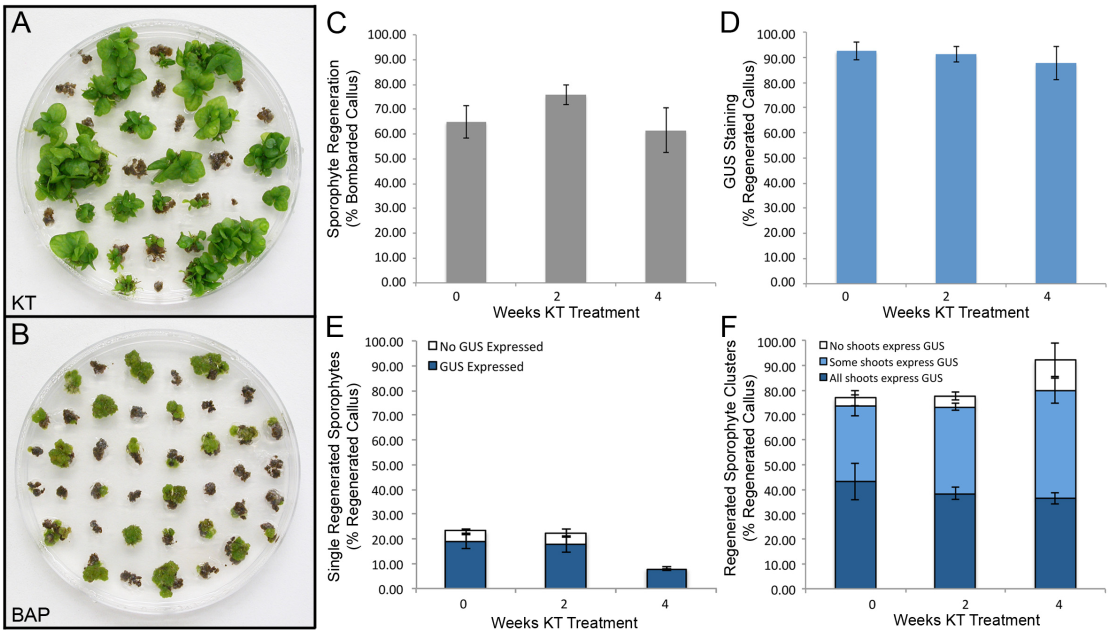

A-F: Optimisation of CK treatment during regeneration of T_0 sporophytes. Bombardment and regeneration on media initially containing 5 µM KT results in successful tissue differentiation by 6 weeks after removal of KT treatment. On media initially containing 5 µM BAP tissue differentiation does not occur, even after the removal of BAP treatment (B). Successful regeneration of transformed sporophytes was achieved even in the absence of KT treatment during regeneration. The effects of altering the length of KT treatment on the regeneration frequency of T_0 sporophytes as a percentage of callus bombarded (C) are shown, as is the percentage of those

regenerated sporophytes expressing GUS (D). The relative frequencies of calli regenerating single sporophytes (E) and clusters of multiple sporophytes (F) under each treatment are shown, along with their associated frequencies of GUS staining.

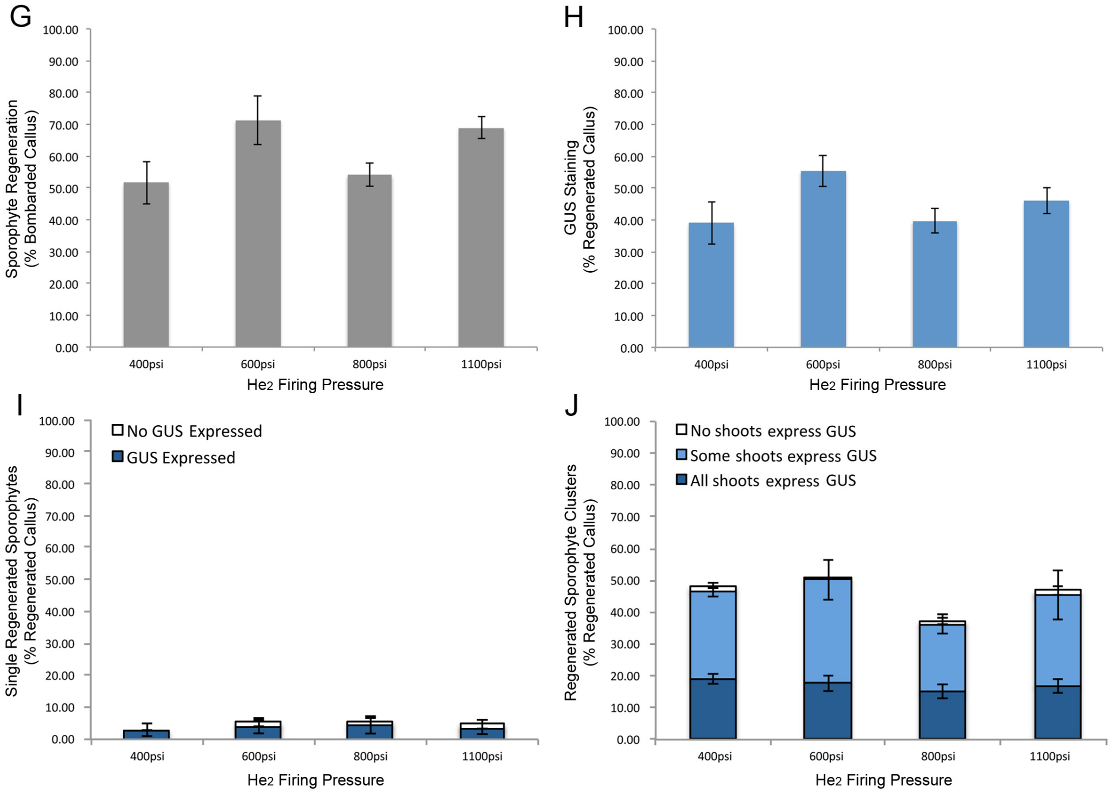

G-J: Optimisation of He_2 firing pressure during particle bombardment.

A range of firing pressures (400-1100 psi) were tested, the choice of pressures within the range being determined by the tolerances of commercially-available rupture discs (Bio-Rad). Successful regeneration of transgenic sporophytes was achieved under all firing pressures tested. The effects of altering firing pressure on the regeneration frequency of T_0 sporophytes as a percentage of callus bombarded (G) are shown, as is the percentage of those regenerated sporophytes expressing GUS (H). The relative frequencies of calli regenerating single sporophytes (I) and clusters of multiple

sporophytes (J) under each treatment are shown, along with their associated frequencies of GUS staining.

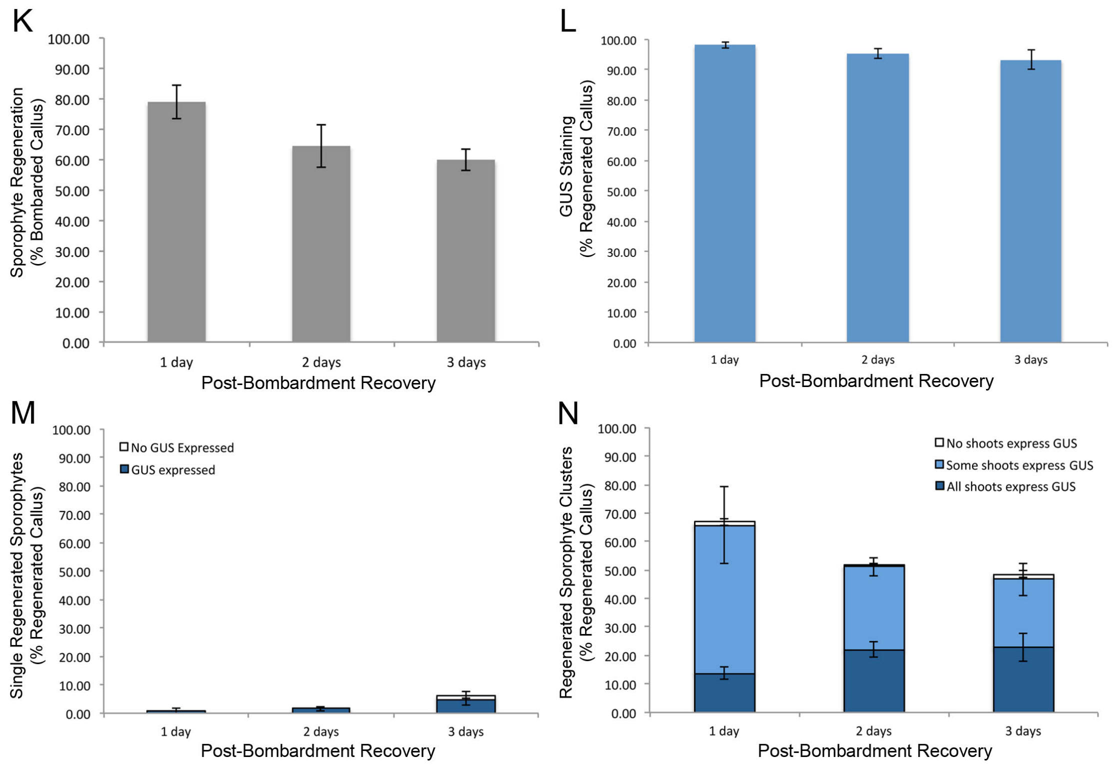

K-N: Optimisation of the length of post-bombardment callus recovery prior to selection.

A recovery interval allows the healing of callus cell walls after bombardment and integration of introduced transgene DNA into the nucleus/genome. Successful regeneration of transgenic sporophytes was achieved across the range of recovery intervals tested (1-3 days). The effects of changing the length of post-bombardment recovery on the regeneration frequency of T_0 sporophytes as a percentage of callus bombarded (K) are shown, as is the percentage of those regenerated sporophytes expressing GUS (L). The relative frequencies of calli regenerating single sporophytes

(M) and clusters of multiple sporophytes (N) under each treatment are shown, along with their associated frequencies of GUS staining.
