# Supplementary material for: Protocol: genetic transformation of the fern Ceratopteris richardii through microparticle bombardment
Source: Plant Methods. 2015 Jul 3;11:37. doi: 10.1186/s13007-015-0080-8 (PMC4490597; doi:10.1186/s13007-015-0080-8)
Supplement: Additional file 7: — Preparation of microparticles for bombardment. Protocols for the sterilisation and DNA-labelling of microparticles for bombardment. [file 13007_2015_80_MOESM7_ESM.pdf]

Additional File 7: Preparation of microparticles for bombardment.

Bombardments used in this protocol were performed using Bio-Rad tungsten M-20 microparticles (average diameter approximately 1.3 µm).

A. Microparticle sterilisation

The volume of microparticles prepared is dependent on the anticipated number of bombardments to be performed: the required final microparticle concentration is 60 mg/ml. The protocol below is specified for 1 ml of sterile microparticles (20 bombardments). For fewer bombardments, reduce the starting amount of microparticles and final resuspension volume accordingly.

1. Weigh out 60 mg of dry microparticles in a 1.5 ml microcentrifuge tube.

2. Add 1 ml freshly-prepared 70% EtOH to microparticles, vortex on a platform vortexer for 5 minutes, then incubate for 15 minutes.

3. Pellet microparticles by centrifuging 13,200 rpm (or maximum speed) for one minute. Remove the supernatant and discard.

4. Add 1 ml sterile water to microparticles and vortex for one minute. Allow microparticles to settle for one minute, then centrifuge 13,200 rpm for one minute. Remove the supernatant and discard.

5. Perform step A4 three times.

6. Add 1 ml sterile 50% glycerol to microparticles to resuspend.

NOTE: It is assumed that no loss of microparticles occurred during sterilisation.

7. Store sterilised microparticles at room temperature.

NOTE: Sterile microparticle stocks can be stored for up to two weeks and still be used for successful bombardments.

B. Microparticle labelling

Labelling of microparticles should be performed on the day of bombardment, preferably immediately prior to bombardment. It is not necessary to prepare microparticles in a laminar flow hood under sterile conditions, but sterile water and tips should be used. The protocol described below is calculated to provide sufficient microparticles for five bombardments with one plasmid. This protocol requires 5 μg of plasmid DNA at a concentration of 1 μg/μl. It is recommended that a No DNA control be prepared at the same time, substituting water for plasmid DNA at step B3.

1. On a platform vortexer, vigorously vortex sterile microparticles for five minutes to resuspend them.

2. Whilst the tube is still being vortexed, using a Gilson pipette withdraw 50 μl resuspended microparticles and place in a clean 1.5 ml microcentrifuge tube. Prepare one aliquot of microparticles per plasmid/No DNA control to be bombarded. The microparticle stock can now be stored.

3. Vortex 50 µl microparticle aliquots on the platform vortexer. Whilst vortexing, add the following reagents in order:

- 5 µl plasmid DNA (1 µg/µl). Substitute sterile water for No DNA control.
- 50 µl $CaCl_2$ (2.5 M, filter-sterilized)
- 20 µl spermidine (100 mM, filter-sterilized)

4. Continue vortexing microparticles for three minutes, then allow microparticles to settle for one minute.

NOTE: In comparison to unlabelled controls, labelled microparticles typically settle more rapidly. The addition of sperimidine can cause microparticles to agglomerate, but this does not appear to significantly affect the success of subsequent bombardments.

5. Centrifuge 13,200 rpm for one minute to pellet microparticles. Remove supernatant and discard.

6. Add 150 µl 70% EtOH to the tube without disturbing the pellet, then remove the liquid and discard.

7. Add 150 µl 100% EtOH to the tube without disturbing the pellet, then remove the liquid and discard. Add 50 µl 100% EtOH to dehydrated microparticles and tap gently to resuspend. Microparticles are now ready for immediate bombardment, using 10 µl per bombardment.

TROUBLESHOOTING: If microparticles fail to resuspend easily, vortex them briefly to disrupt agglomerations. If vortexing fails to disperse microparticles evenly, manually break up clumps using a pipette whilst dispensing them.
