# Supplementary material for: Protocol: genetic transformation of the fern Ceratopteris richardii through microparticle bombardment
Source: Plant Methods. 2015 Jul 3;11:37. doi: 10.1186/s13007-015-0080-8 (PMC4490597; doi:10.1186/s13007-015-0080-8)
Supplement: Additional file 8: — Direct induction of transgenic sporophytes from gametophytes under antibiotic selection. An example of the successful generation of T1 sporophytes from gametophytes under antibiotic selection and subsequent confirmation of their transgenic status. [file 13007_2015_80_MOESM8_ESM.pdf]

Additional File 8: Direct induction of transgenic sporophytes from gametophytes under antibiotic selection.

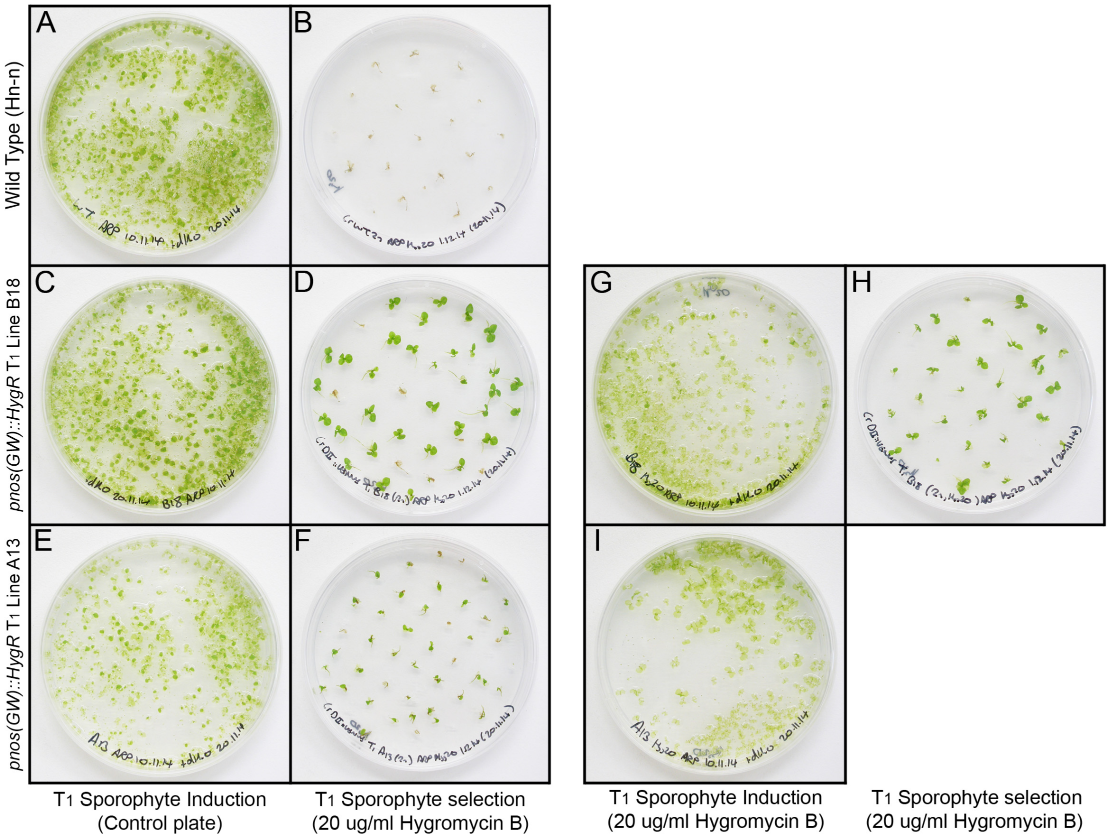

Successful generation of T_1 sporophytes from antibiotic-resistant T_1 gametophytes was observed when hygromycin resistance was driven by the pnos(GW) promoter. Adding water to the control wild-type gametophyte plate successfully induced fertilization and subsequent sporophyte development (A). Control plates for T_1 lines also successfully generated sporophytes in the absence of hygromycin B (C, E). Successful antibiotic selection was confirmed by the death of WT sporophytes on selection (B), whilst sporophytes induced from T_1 gametophytes in the absence of selection contained a mixed population of resistant and susceptible sporophytes (D, F).

Sporophyte induction from hygromycin-resistant T_1 gametophytes was attempted by adding water to selective gametophyte plates, still in the presence of hygromycin selection. In some T_1 lines (e.g. B18) sporophytes were successfully induced (G). Subsequent antibiotic resistance screening of these sporophytes confirmed antibiotic resistance in all sporophytes tested (H). However, in sibling T_1 lines (e.g. A13) the attempted induction of sporophytes under antibiotic selection failed (I).
